# Epipericardial fat necrosis in chest CT and MRI: a case report of an unusual cause of chest pain associated with the initial diagnosis of undifferentiated connective tissue disease

**DOI:** 10.1186/s12872-023-03349-x

**Published:** 2023-06-22

**Authors:** Inês Barreto, Francisca Godinho Oliveira, Sofia Carvalho Barreira, João Rodrigues Inácio

**Affiliations:** 1 Pulmonology Department, North Lisbon University Hospital Centre (CHULN), Lisbon Medical Academic Centre (CAML), Avenida Professor Egas Moniz 1649-035, Lisbon, Portugal; 2 Rheumatology Department, North Lisbon University Hospital Centre (CHULN), Lisbon Medical Academic Centre (CAML), Lisbon, Portugal; 3 Rheumatology Research Unit, Institute of Molecular Medicine (IMM), Faculty of Medicine of the University of Lisbon, Lisbon Medical Academic Centre (CAML), Lisbon, Portugal; 4 Radiology Department, North Lisbon University Hospital Centre (CHUNL), Lisbon Medical Academic Centre (CAML), Lisbon, Portugal; 5 Radiology Clinic, Faculty of Medicine of the University of Lisbon, Lisbon Medical Academic Centre (CAML), Lisbon, Portugal

**Keywords:** Epipericardial fat necrosis, Chest pain, CT, MRI, Undifferentiated connective tissue disease

## Abstract

**Background:**

Epipericardial fat necrosis (EFN) is a benign and self-limited condition of unknown cause with a good prognosis, usually affecting otherwise healthy patients. Clinically, it presents with severe acute left pleuritic chest pain, often leading the patient to the Emergency Room (ER).

**Case presentation:**

A 23-year-old male, smoker (5 pack-years), was evaluated in the ER due to left pleuritic chest pain, worsening with deep breathing and Valsalva maneuver. It was not associated with trauma and did not present other symptoms.

The physical examination was unremarkable. The arterial blood gases while breathing room air and the laboratory tests, including D-dimers and high-sensitivity cardiac Troponin T, were normal. The chest radiograph, electrocardiogram, and transthoracic echocardiogram showed no abnormalities.

A computed tomography (CT) pulmonary angiogram showed no signs of pulmonary embolism but depicted at the left cardiophrenic angle a focal 3 cm ovoid-shaped fat lesion with stranding and thin soft tissue margins, consistent with necrosis of the epicardial fat, which was confirmed by magnetic resonance (MRI) of the chest.

The patient was medicated with ibuprofen and pantoprazole, with clinical improvement in four weeks. At a two-month follow-up, he was asymptomatic and presented radiologic resolution of the inflammatory changes of the epicardial fat of the left cardiophrenic angle on chest CT. Laboratory tests revealed positive antinuclear antibodies, positive anti-RNP antibody, and positive lupus anticoagulant. The patient complained of biphasic Raynaud’s phenomenon initiated five years ago, and a diagnosis of undifferentiated connective tissue disease (UCTD) was made.

**Conclusions:**

This case report highlights the diagnosis of EFN as a rare and frequently unknown clinical condition, which should be considered in the differential diagnosis of acute chest pain. It can mimic emergent conditions such as pulmonary embolism, acute coronary syndrome, or acute pericarditis. The diagnosis is confirmed by CT of the thorax or MRI. The treatment is supportive and usually includes non-steroidal anti-inflammatory drugs. The association of EFN with UCTD has not been previously described in the medical literature.

**Supplementary Information:**

The online version contains supplementary material available at 10.1186/s12872-023-03349-x.

## Background

Epipericardial fat necrosis (EFN), also known as pericardial fat necrosis or epicardial fat necrosis, is a rare and self-limiting condition usually occurring in otherwise healthy patients. It presents with acute pleuritic chest pain, which can mimic emergent conditions such as pulmonary embolism, acute coronary syndrome, or acute pericarditis [1–4].

The diagnosis can be made with chest computed tomography (CT), which typically demonstrates a round or ovoid encapsulated mediastinal fat-containing lesion in the cardiophrenic space, with thin soft tissue density margins [2, 5]. On magnetic resonance (MRI), there is a focal fatty lesion with increased signal in T2 fluid sensitive sequences in the acute phase [5].

The treatment is usually supportive and includes non-steroidal anti-inflammatory drugs and the prognosis is good [1, 6].

## Case presentation

The authors present a case of a 23-year-old Caucasian male, current smoker with five pack-years smoking history, with sporadic consumption of inhaled cannabis. He had a history of bilateral tonsillectomy and septoplasty.

The patient presented to the Emergency Room (ER) complaining of severe acute left pleuritic chest pain, worsening with deep inspiration and Valsalva maneuver. There was no history of chest trauma, fever, cough, sputum, wheezing, hemoptysis, or constitutional symptoms. He did not present any recent illness in the month that preceded the current complaint.

On physical examination, he was apyretic, eupneic breathing room air, with peripheral oxygen saturation of 96%, blood pressure 102/54 mmHg, and heart rate of 77 bpm. Cardiac and pulmonary auscultation were normal. The patient’s body mass index (BMI) was 19.26 kg/m^2^ (weight: 71.0 kg; height: 1.92 m).

The arterial blood gases while breathing room air and the laboratory tests (including the complete blood count, kidney function, ionogram, and liver enzymes) were normal, being of note: D-dimers 0,37 ug/mL [normal range (NR): 0.0-0.5 ug/mL]; high-sensitivity cardiac Troponin T (hs-cTnT) < 3 ng/L [NR: < 14 ng/L]; c-reactive protein (CRP) 0.470 mg/dL [NR: < 0.5 mg/dL]; erythrocyte sedimentation rate (ESR) 4 mm [NR: < 10 mm]. The viral panel, including human immunodeficiency virus (HIV), coxsackievirus, cytomegalovirus, Epstein-Barr virus, and hepatitis B and C virus, was negative. The chest radiograph presented no pulmonary consolidation, pneumothorax, or pleural effusion. The electrocardiogram presented sinus rhythm, heart rate of 65 bpm, with no signs of myocardial ischemia. The transthoracic echocardiogram showed normal size of the left ventricle, normal wall thickness, normal left ventricular function, with no regional wall motion abnormalities, no valvular abnormalities, no pericardial effusion or thickening, and no evidence of acute or chronic myocarditis.

**Fig. 1:**
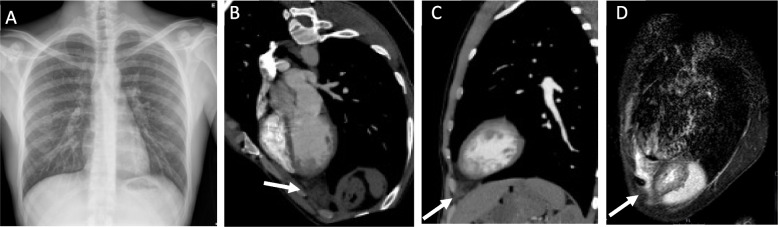
**A** – Normal chest radiograph; **B** and **C** – Contrast-enhanced chest CT reformatted axial and sagittal oblique images of the mediastinum demonstrate an ovoid-shaped mediastinal fat lesion with soft tissue stranding, consistent with necrosis of the epicardial fat at the left cardiophrenic angle (white arrows). **D** – Chest MRI sagittal oblique fluid sensitive T2 sequence image shows a fatty lesion with increased signal (edema) stranding in the left cardiophrenic angle (white arrow)

A CT pulmonary angiogram showed no pulmonary embolism or vascular abnormalities. However, it depicted at the left cardiophrenic angle a focal 3 cm ovoid-shaped fat lesion with stranding and thin soft tissue margins, consistent with necrosis of the epicardial fat (Fig. 1A-C and Supplementary Material – Files 1, 2 and 3). The lungs, the pleura and the pericardium were normal.

Chest MRI demonstrated a 3 cm fatty lesion with increased T2 signal in the left cardiophrenic angle consistent with focal edema (Fig. 1D and Supplementary Material – Files 4 and 5).

**Fig. 2:**
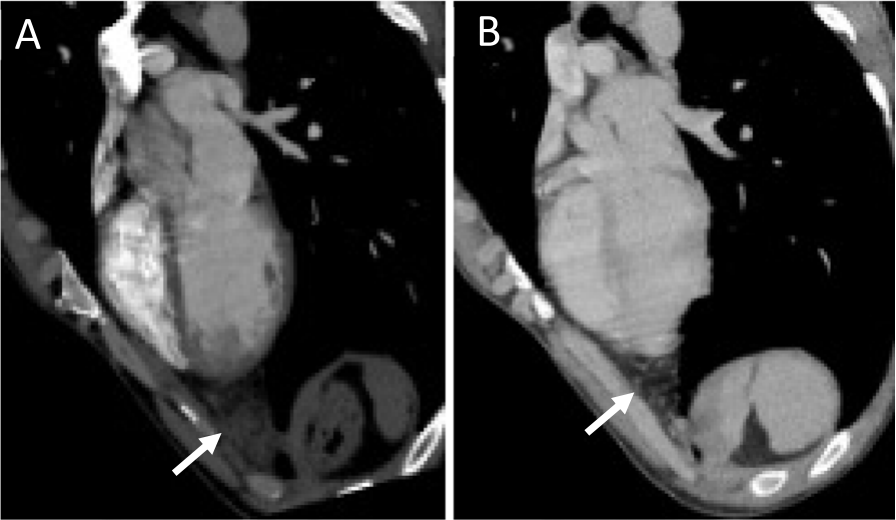
Comparison of contrast enhanced chest CT at the time of clinical presentation (**A**) and after two months (**B**). Reformatted axial oblique images demonstrating resolution of the epicardial fat stranding of the left cardiophrenic angle (white arrows)

The patient was medicated with ibuprofen 600 mg twice daily and pantoprazole 40 mg once daily, with symptomatic improvement after four weeks. A chest CT follow-up was performed two months after and confirmed the resolution of the inflammatory changes of the epicardial fat of the left cardiophrenic angle (Fig. 2B).

Laboratory tests at the two-month follow-up appointment revealed positive antinuclear antibodies (titer of 1/1280, with a speckled nuclear pattern, AC-5) and a positive anti-RNP antibody (> 6438.0 UQ). In addition, the lupus anticoagulant was positive, with a negative anticardiolipin and negative anti-beta-2-glycoprotein. All antiphospholipid antibodies were negative at the 12-week reassessment. The ESR, CRP, creatine phosphokinase, aldolase, complement C3 and C4 fractions, immunoglobulins and the urine analysis were normal.

Lung function tests presented normal spirometry, plethysmography, and alveolo-capillary diffusing capacity for carbon monoxide.

When further asked about other current or previous systemic symptoms, the patient complained about biphasic Raynaud’s phenomenon initiated five years ago. He had no history of digital or oral ulcers, arthralgias, myalgias, muscle weakness, rash, photosensitivity, alopecia, dysphagia, dyspnea, or fever.

**Fig. 3:**
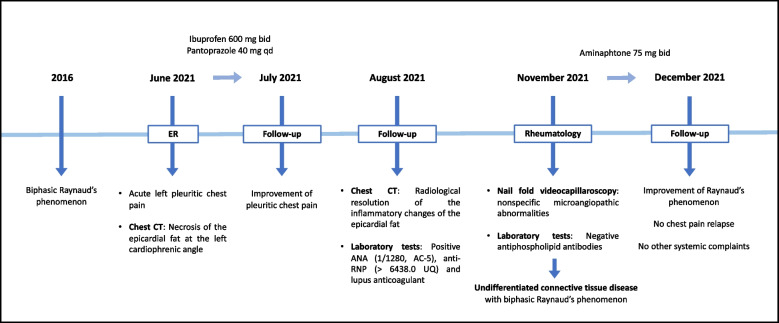
Timeline highlighting the patient’s clinical presentation and most relevant complementary exams findings

The patient was referred to a Rheumatology consult to assess a possible mixed connective tissue disease (MCTD). The nail fold videocapillaroscopy presented nonspecific microangiopathic abnormalities. The chest CT images did not show interstitial lung disease (ILD). A diagnosis of undifferentiated connective tissue disease (UCTD) with biphasic Raynaud’s phenomenon was made, and the patient was medicated with aminaphtone 75 mg twice daily with clinical improvement of Raynaud’s phenomenon. The patient is under follow-up for other manifestations such as synovitis and myositis, which could mean a differentiation into a MCTD in a patient with high anti-RNP antibody titers. A timeline highlighting the patient’s clinical presentation and most relevant complementary exams findings is presented in Fig. 3.

## Discussion and conclusions

This case highlights the diagnosis of EFN as a rare and frequently unknown medical condition, which should be considered in the differential diagnosis of acute chest pain in otherwise healthy patients [1].

Initially described in 1957 by Jackson et al. [7], EFN is thought to be related to an inflammatory process in the mediastinal fat surrounding the heart, affecting equally males and females [1]. It presents as severe acute pleuritic chest pain, ipsilateral to the lesion, usually on the left side, which may radiate to the neck, shoulder, upper arm, axilla, or back [4]. EFN can present with syncope, tachycardia and dyspnea that can mimic emergent conditions frequently leading the patient to the ER, such as pulmonary embolism, acute coronary syndrome, acute aortic syndrome, or acute pericarditis [1, 4, 8]. Cough and fever have never been reported in association with EFN [4].

A pericardial friction rub may be present on physical examination, and some patients may have tenderness to palpation over or near the precordium [1, 4]. The laboratory tests, including inflammatory markers, myocardial necrosis markers, D-dimer, and the electrocardiogram (ECG), are usually normal, and the viral panel is negative [1]. Occasionally, the ECG may show tachycardia and nonspecific ST or T-wave changes [4]. Transthoracic and transesophageal echocardiography may show an ovoid, solid pericardial mass [4].

The chest radiograph is nonspecific and is usually normal in the first 48-72 hours, after which there may be seen a juxtacardiac opacity near the cardiophrenic angle on the side of the chest pain, located anteriorly and usually merging with the cardiac silhouette, with or without a pleural effusion [1, 2, 8]. Chest CT is the preferred modality for diagnosing EFN, localising the abnormality in cardiophrenic space, and density measurements allowing confident characterisation of abnormal epicardial fat [1, 2, 5]. Chest CT typically demonstrates a round or ovoid encapsulated mediastinal fat-containing lesion with thin soft tissue density margins, and inflammatory changes of fat stranding and thickening of the adjacent epicardium [2]. Pleural effusion and adjacent subsegmental lung atelectasis can also be identified at the time of diagnosis [2, 4]. Findings on MRI include a focal fatty lesion with increased signal in T2 fluid sensitive sequences in the acute phase [5]. Gallium scintigraphy may show increased gallium uptake in the pericardial fat [4].

The treatment is usually supportive and includes non-steroidal anti-inflammatory drugs. The pain is usually severe, may persist for several weeks, and can recur with less intensity for up to a year, but the prognosis is good [1, 6].

The differential diagnosis includes pericardial lipoma, pericardial liposarcoma, thymolipoma, and diaphragmatic hernia. Although the pathogenesis of EFN is unclear, some predisposing factors have been identified, such as trauma, ischemia related to acute epicardial fat torsion, high positioned pericardial fat, obesity, hemorrhagic necrosis due to increased thoracic pressure related to Valsalva maneuver, pre-existing structural abnormality of the adipose tissue, such as lipoma or hamartoma [1, 4, 8]. The association of EFN with rheumatologic conditions has not been previously established in the scientific literature.

The density values on CT and MRI signal characteristics allow a confident diagnosis of a fat lesion with edema in the acute setting [4]. If there is persistence of symptoms, persistence or growth of soft tissue and fat abnormalities on follow-up imaging studies, it may be impossible to distinguish between a benign and malignant fatty tumor, in which case surgical intervention may be indicated to confirm the diagnosis [4]. Some patients underwent exploratory thoracotomy after extensive nondiagnostic workups, presenting an inflammatory mass involving the parietal pericardial fat, whose histopathology showed resemblance to analogous conditions, such as epiploic appendagitis and fat necrosis in the omentum or breast [4]. In early phases, there is a central focus of necrotic fat cells surrounded by macrophages with neutrophilic infiltration, and in latter phases, there is also marked fibrosis. Calcifications may be seen. Removal of the lesion always leads to remission of symptoms [1, 4]. However, considering the characteristic clinical and radiological presentation and its benign course, a clinical diagnosis and supportive treatment should be privileged, reserving thoracic surgery only when the patient presents severe untreatable pain or unusual imaging findings [2, 4]. A follow-up chest CT or MRI 4 to 8 weeks after the acute presentation is recommended to confirm the expected healing [1, 2, 4].

Although only a few cases of EFN have been published in the literature, suggesting it is an infrequent diagnosis, recent case series suggest that it might be more prevalent than previously thought and may frequently be underdiagnosed [1, 2]. It is considered a benign self-limited condition, but in our case, EFN presented in a patient subsequently diagnosed with UCTD, which, to our current knowledge, has not been previously described, warranting clinical awareness of this potential association in this population. Rheumatologic conditions such as systemic lupus erythematosus (SLE) and UCTD have been linked to an increased risk of thrombosis, to which factors such as inflammation, endothelial dysfunction, and antiphospholipid antibodies, especially lupus anticoagulant, play a significant role [9–17]. The authors hypothesize that this prothrombotic status might be a predisposing cause leading to EFN in this patient, although further research is needed.

## Supplementary Information

**Additional file 1.** Contrast-enhanced chest CT axial, sagittaland reformatted sagittal obliqueclip videos demonstrating an ovoid-shaped mediastinal fat lesion with soft tissue stranding, consistent with necrosis of the epicardial fat at the left cardiophrenic angle.

**Additional file 2.** Chest MRI sagittal (File 4) and sagittal oblique (File 5) fluid sensitive T2 sequence clip videos showing a fatty lesion with increased signal (edema) stranding in the left cardiophrenic angle.

## Data Availability

All data and materials are available for sharing if needed. The corresponding author should be contacted for access to the data.

## Abbreviations

BMI: Body mass index
CRP: C-reactive protein
CT: Computed tomography
ECG: Electrocardiogram
EFN: Epipericardial fat necrosis
ER: Emergency room
ESR: Erythrocyte sedimentation rate
HIV: Human immunodeficiency virus
hs-cTnT: High-sensitivity cardiac Troponin T
ILD: Interstitial lung disease
MCTD: Mixed connective tissue disease
MRI: Magnetic resonance
NR: Normal range
NT-proBNP: N-terminal pro b-type natriuretic peptide
SLE: Systemic lupus erythematosus
UCTD: Undifferentiated connective tissue disease
